# Transcriptomic Analysis of Streptococcus pyogenes Colonizing the Vaginal Mucosa Identifies *hupY*, an MtsR-Regulated Adhesin Involved in Heme Utilization

**DOI:** 10.1128/mBio.00848-19

**Published:** 2019-06-25

**Authors:** Laura C. C. Cook, Nilanjana Chatterjee, Yan Li, Jorge Andrade, Michael J. Federle, Zehava Eichenbaum

**Affiliations:** a Binghamton Biofilm Research Center, Department of Biology, Binghamton University, Binghamton, New York, USA; b Department of Biology, Georgia State University, Atlanta, Georgia, USA; c Center for Research Informatics, The University of Chicago, Chicago, Illinois, USA; d Department of Medicinal Chemistry and Pharmacognosy, University of Illinois at Chicago, Chicago, Illinois, USA; e Center for Research Informatics and Department of Pediatrics, Biological Sciences Division, The University of Chicago, Chicago, Illinois, USA; KUMC

**Keywords:** colonization, *Streptococcus*, adherence, heme, iron uptake

## Abstract

Streptococcus pyogenes (group A streptococcus [GAS]) is a serious human pathogen with the ability to colonize mucosal surfaces such as the nasopharynx and vaginal tract, often leading to infections such as pharyngitis and vulvovaginitis. We present genome-wide transcriptome sequencing (RNASeq) data showing the transcriptomic changes GAS undergoes during vaginal colonization. These data reveal that the regulon controlled by MtsR, a master metal regulator, is activated during vaginal colonization. This regulon includes two genes highly expressed during vaginal colonization, *hupYZ*. Here we show that HupY binds heme *in vitro*, affects intracellular concentrations of iron, and is essential for proper growth of GAS using hemoglobin or serum as the sole iron source. HupY is also important for murine vaginal colonization of both GAS and the related vaginal colonizer and pathogen Streptococcus agalactiae (group B streptococcus [GBS]). These data provide essential information on the link between metal regulation and mucosal colonization in both GAS and GBS.

## Abstract (precis)

Colonization of the host requires the ability to adapt to an environment that is often low in essential nutrients such as iron. Here we present data showing that the transcriptome of the important human pathogen Streptococcus pyogenes shows extensive remodeling during *in vivo* growth, resulting in, among many other differentially expressed genes and pathways, a significant increase in genes involved in acquiring iron from host heme. Data show that HupY, previously characterized as an adhesin in both S. pyogenes and the related pathogen Streptococcus agalactiae, binds heme and affects intracellular iron concentrations. HupY, a protein with no known heme binding domains, represents a novel heme binding protein playing an important role in bacterial iron homeostasis as well as vaginal colonization.

## INTRODUCTION

Streptococcus pyogenes (group A streptococcus [GAS]) is an important primary pathogen causing severe infections like necrotizing fasciitis and toxic shock syndrome, but it also colonizes mucosal surfaces, often asymptomatically. Mucosal carriage of GAS in the throat (1–3), gastrointestinal tract (4), and rectovaginal tract (5, 6) can serve as principal reservoirs for community infections. Although the rate of transmission from carriers is lower than in acutely infected individuals, this reservoir is important on a population level, as rates of carriage greatly eclipse rates of acute infections in the community (7).

Vaginal mucosal colonization by GAS is associated with vulvovaginitis in prepubertal girls, with studies reporting that 11 of 20% of swabs collected from girls with vulvovaginitis contained GAS (8–10). A rectovaginal carrier state has been demonstrated in adult women (6, 11), and although the level of vaginitis is lower in adults, it has been reported in the literature (4, 12). A murine vaginal colonization model has been developed for GAS based on a similar model used for the related Streptococcus agalactiae (group B streptococcus [GBS]) (13–15). This model not only allows for examination of GAS vaginal colonization but also provides an easily accessible model for colonization of host mucosal surfaces. Here we describe the transcriptome of GAS during murine vaginal carriage. This work, in conjunction with previous research describing transcriptional profiles during vaginal colonization by GBS (16), provides an important framework for the genetic changes streptococcal pathogens undergo during mucosal carriage.

The environment encountered in mucosal surfaces is vastly different from liquid laboratory culture, which is reflected in the large number of genetic changes observed via transcriptome sequencing (RNASeq). One set of genes that was highly differentially expressed during GAS vaginal colonization is known to be under the regulation of MtsR (Spy49_0380c), a master regulator of iron homeostasis and virulence in GAS and related streptococci (17–19). Under iron-replete conditions, MtsR acts as a negative regulator of over 40 genes in GAS, including the ribonucleotide reductase operon *nrdF.2IE.2* operon (*spy49_0339-0341*) and many genes involved in heme metabolism, such as *shr* (*spy49_1405c*), *shp* (*spy49_1404c*), *siaABC* (*spy49_1401c-1403c*), and *hupZ* (*spy49_0662*) (18, 20, 21).

HupZ was recently described as a novel enzyme involved in GAS heme biotransformation. HupZ binds and degrades heme to generate free iron *in vitro* (20). As a cytoplasmic enzyme, HupZ does not have access to extracellular heme and thus depends on GAS uptake machinery for heme supply. Heme acquisition in Gram-positive bacteria typically involves surface receptors that capture heme from the host and deliver it through the peptidoglycan layers to dedicated ABC transporters in the membrane for import into the cytoplasm (22). The only receptors for hemoproteins and heme described for GAS are Shr and Shp, which together consists of a heme relay system that shuttles heme from the extracellular environment to the SiaABC heme transporter (also known as *htsABC*) (23–25). Here we report that the gene adjacent to the HupZ gene, *spy49_0661* (now renamed the HupY gene), is highly upregulated during vaginal carriage and not only is important for mucosal colonization but also plays a role in heme utilization in GAS.

HupY, previously known as LrrG, is a leucine-rich repeat protein with homologs in other species of streptococci, including GBS (SAK_0502). These proteins have previously been described as LPXTG-anchored cell surface proteins in GAS and GBS that are involved in binding epithelial cells. Immunization against LrrG was protective in a mouse model of GAS infection, and it was also shown to be expressed during a macaque model of acute pharyngitis (26–28). Genetic location and coregulation indicate that the functions of HupZ and HupY may be related. We hypothesize that HupY serves as both an adhesin and a receptor that facilitates the capture and uptake of heme into GAS during colonization and infection of the host.

## RESULTS

### Extensive transcriptional remodeling of GAS takes place during murine vaginal colonization.

**FIG 1:**
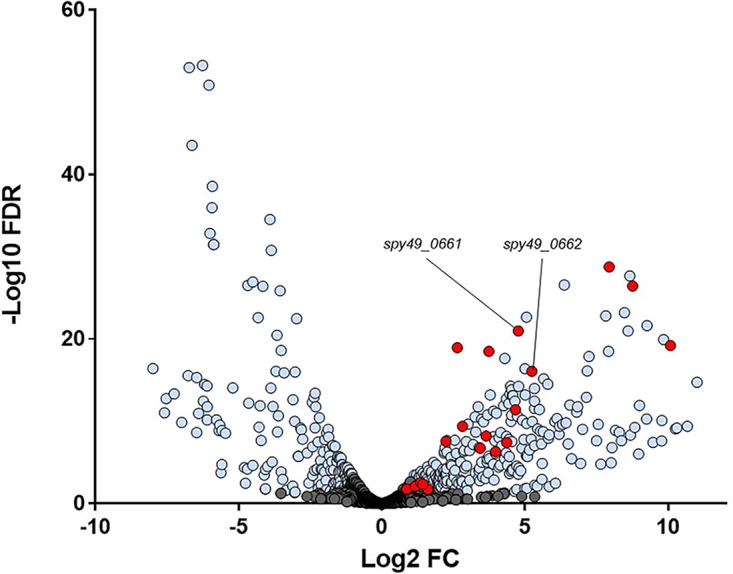
Differentially expressed genes during GAS growth *in vivo*. The volcano plot shows transcriptomic changes in GAS genes during growth in liquid versus colonizing the murine vaginal tract with –log_10_ false-discovery rate (FDR) adjusted *P* value on the *y* axis and log_2_ fold change during murine vaginal growth on the *x* axis. Genes in blue were significantly differentially expressed with an FDR adjusted *P* value of <0.05 and a fold change of >2. Genes known to be regulated by the metal regulator MtsR are shown in red, and *hupY* (*spy49_0661*)/*hupZ* (*spy49_0662*) are specifically marked.

Mice were vaginally inoculated with GAS strain NZ131, and after 48 h of colonization, vaginal lavage samples containing GAS cells were collected for RNASeq analysis. Vaginal carriage samples were compared to log-phase NZ131 bacteria grown statically at 37°C in chemically defined laboratory medium (CDM). Of the 1,686 NZ131 genes with transcripts detected, 581 genes were differentially expressed, with a false-discovery rate (FDR) corrected *P* value of ≤0.05. Of those, 491 genes had a fold change of ≥2 (Fig. 1; see also Table S1 in the supplemental material). To validate RNASeq findings, qualitative real-time PCR was done on two genes shown to be downregulated in the vaginal tract (Fig. S1).

10.1128/mBio.00848-19.1 FIG S1 qRT-PCR data validated the RNASeq results. Quantitative RT-PCR was done on two genes shown to be downregulated in the vaginal tract via RNASeq (*spy49_1781* and *spy49_1782*). qPCR showed that both genes were downregulated in the vaginal tract (darker bars), matching the expression patterns obtained by RNA sequencing (lighter bars). Download FIG S1, TIF file, 0.04 MB. Copyright © 2019 Cook et al. 2019 Cook et al. This content is distributed under the terms of the Creative Commons Attribution 4.0 International license.

10.1128/mBio.00848-19.3 TABLE S1 Differentially expressed genes in the murine vaginal tract. Genes that were differentially expressed in the vaginal tract are listed with their respective fold change relative to growth in liquid medium (CDM) as well as their false-discovery rate (FDR). Download Table S1, XLSX file, 0.05 MB. Copyright © 2019 Cook et al. 2019 Cook et al. This content is distributed under the terms of the Creative Commons Attribution 4.0 International license.

Like the previously published transcriptome of GBS in the murine vaginal tract (16), there are extensive changes in metabolic pathways associated with host colonization in GAS. Similar to GBS, GAS cells significantly downregulated the fatty acid biosynthesis *fab* operon (*spy49_1359c-1371c*), ostensibly resulting in increased long-chain fatty acids. In other species of streptococci, including Streptococcus gordonii and Streptococcus salivarius, an increase in long-chain and monounsaturated fatty acids was observed in response to acidic pH and hypothesized to be an adaptive response to acidic environments (16, 29) (Table S1).

Both GAS and GBS also highly upregulated homologous phosphotransferase systems essential for the import of a variety of sugars such as ascorbate (*spy49_0154* and *-0155* and *sak_1833* and *-1834*), mannose (*spy49_0834* to -*0836* and *sak_1908* to -*1910*), maltose (*spy49_1028* to -*1030* and *sak_1475* to -*1477*), and galactose and fructose (*spy49_1327c* to -*1329c* and *sak_1893* to -*1895*). Genes and operons involved in degradation of sugars such as ascorbate (*spy49_0156* to -*0158* and -*0160* and *sak_1820* and -*1830* to -*1832*), galactose (*spy49_0673*, -*1322c* to -*1323c*, -*1325*, and -*1326c* and *sak_0345* and -*346* and -*1878* to -*1890*), and malate (*spy49_0864* to -*0865c* and *sak_1651* and -*1878*) were often also upregulated in both organisms (Table S1) (16).

Not all homologous metabolic pathways were similarly regulated between GAS and GBS. During GBS colonization, genes involved in the *de novo* purine biosynthetic pathway *purCLFMNH* (*sak_0057*, *sak_0059-0061*, and *sak_0063*) and *purDEKB* (*sak_0076* to -*0078* and *sak_0080*) were upregulated between 2- and 25-fold (16). Interestingly, the purine biosynthesis pathway in GAS (*spy49_0020* to -*0025*, *spy49*_0027 to -*0029*, and *spy49_31*) was significantly downregulated during colonization compared with log-phase growth in the same medium (Table S1). Although GAS and GBS are closely related species that both colonize mucosal surfaces, their metabolic profiles, while similar, demonstrate some distinct transcriptional changes associated with vaginal colonization. Growth phase is an important factor determining transcriptional profiles. Growth in the vaginal tract does not equate to log-phase growth in liquid culture, which likely explains some of the observed large-scale transcriptional shifts. Some genes highly upregulated during stationary phase in liquid culture are also upregulated in the vaginal tract. *speB* and *mf*, for example, are known to be upregulated during stationary-phase liquid growth and expressed at lower levels during other phases of growth (30). In the vaginal tract, compared to log-phase growth, *speB* and *mf* expression is highly upregulated (Table S1). In contrast, there are also genes, like *clpE*, that have previously been shown to be upregulated during stationary phase compared to log-phase growth (31) but are downregulated during growth in the vaginal tract compared to liquid growth (Table S1). This indicates that cells colonizing mucosal surfaces cannot be characterized by typical liquid growth phases. This was previously observed for mucosal colonization in GBS as well, where colonization of the vaginal tract showed some genetic signatures of stationary-phase growth but some characteristics of different phases of liquid growth (16).

### The MtsR regulon of both GAS and GBS is strongly influenced by host carriage.

Notably, some of the most highly upregulated genes during vaginal carriage include those previously shown to be directly regulated by MtsR. MtsR regulates genes involved in iron and heme metabolism, and this regulation is dependent on the concentration of iron and manganese. The MtsR regulon includes the metal transporter *mtsABC* (*spy49_1554* to -*1556*) and the 10 gene streptococcal iron acquisition locus (*sia*), which includes *shr* (*spy49_1405c*), *shp* (*spy49_1404c*), and *siaA-siaH* (*spy49_1395c* to -*1398c* and -*1400* to -*1403c*) (17, 32), all of which are significantly upregulated between 2.5- and >25-fold during growth in the vaginal tract (Table 1 and Fig. 1). Annotation of NZ131 incorrectly lists *siaA* and *shp* as a single fused gene product, although they are actually separate genes (21). Because of the annotation error, gene upregulation for *shp* and *siaA* were measured together over the course of the single transcript which was highly upregulated. Because the genes are separate but coregulated, upregulation of *shp* is inferred from RNASeq data.

**TABLE 1:** Genes repressed by MtsR with differential regulation in the vaginal tract

Gene	Name	Description	Fold change^*a*^	*P* value^*b*^
spy49_0247	pbp7	d-Alanyl–d-alanine carboxypeptidase	2.4	0.00565
spy49_0339	nrdF.2	Ribonucleotide reductase	432.03	3.60E−27
spy49_0340	nrdI	Ribonucleotide reductase stimulatory protein	1079.15	6.20E−20
spy49_0341	nrdE.2	Ribonucleotide diphosphate reductase	245.77	1.80E−29
spy49_0381	mtsA	Metal ABC transporter, substrate binding	6.23	1.10E−19
spy49_0382	mtsB	Metal ABC transporter, ATP binding	1.83	0.00791
spy49_0383	mtsC	Metal ABC transporter, permease	2.24	0.0007
spy49_0661	hupY	Adhesion and heme receptor	27.26	1.10E−21
spy49_0662	hupZ	Enzyme, heme degradation	37.69	8.40E−17
spy49_1159c	manB	Phosphoglucomutase, phosphomannomutase	13.35	3.20E−19
spy49_1374c	dnaK	Chaperone protein	3.05	0.01803
spy49_1395c	siaH	ECF transporter, A component	2.56	0.00283
spy49_1396c	siaG	ECF transporter, T component	2.7	0.00464
spy49_1397c	siaF	ECF transporter, S component	4.71	2.70E−08
spy49_1398c	siaE	Putative ABC transporter	7.03	4.10E−10
spy49_1400c	siaD	Putative ABC transporter	12.49	6.10E−09
spy49_1401c	siaC	Heme ABC transporter, ATP binding	15.68	4.90E−07
spy49_1402c	siaB	Heme ABC transporter, permease	10.82	1.90E−07
spy49_1403c/ 1404c^*c*^	siaA/shp	Heme ABC transporter, substrate binding/streptococcal heme-associated protein	20.58	3.80E−08
spy49 _1405c	shr	Streptococcal hemoprotein receptor	25.46	4.10E−12
spy49_1687c	prsA	Foldase	144.61	7.43E−17

a Fold upregulation in the vaginal tract compared to liquid growth.

b False discovery rate-corrected *P* value.

c Annotation of NZ131 incorrectly lists *spy49_1403c* (*siaA*) and *spy49_1404c* (*shp*) as a single fused gene product rather than two distinct genes.

In addition, MtsR has been shown previously to directly repress genes such as the *nrdF.2IE.2* operon (*spy49_0339* to -*0341*), which is upregulated over 200-fold during vaginal colonization (Table 1 and Table S1), but does not regulate the related *nrdHEF* genes which are slightly repressed in the vaginal tract (18). In total, at least 20 genes, about half of those previously shown to be repressed by MtsR, were significantly upregulated in our RNASeq data (Table 1).

**FIG 2:**
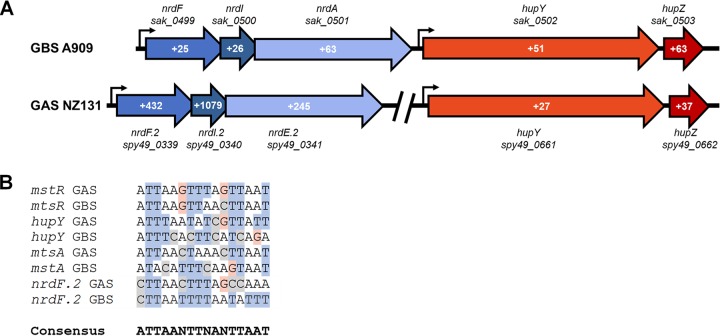
Differential regulation of MtsR-regulated operons in GAS and GBS and MtsR-regulated promoter alignment. (A) Select MtsR-regulated operons of GAS and GBS are shown. Red genes are *hupYZ* homologs and blue genes are *nrdF.2I.2E.2* homologs. Fold change during *in vivo* growth is shown in white. Values from GBS A909 were previously reported (16). (B) Promoter alignment of select MtsR-regulated genes in both GAS and GBS.

Mining previously published transcriptomic data shows that GBS homologs of genes in the GAS MtsR regulon, including *mstABC* (*sak_1554* to -*1556*) and *nrdF.2IE.2* (*sak_499* to -*501*), were similarly upregulated during vaginal colonization and contain putative MtsR promoter DNA binding sites (Fig. 2B) (16). Consensus promoter sequences match previously published known MtsR binding sites (18). It is likely that the MtsR regulon in GBS is similar to that in GAS, and previous transcriptomic data (16) as well as this study suggest that the regulon is derepressed during streptococcal host colonization (Table 1 and Fig. 1).

Transcription of MtsR-regulated *spy49_0662*, also known as *hupZ*, and the gene immediately upstream, *spy49_0661*, are both increased during vaginal colonization (Fig. 1 and 2A and Table 1). Previous RNASeq data from GBS strain A909 colonizing the vaginal tract also showed significant upregulation of the homologous *hupYZ* gene pair (16), and a putative MtsR binding site is located upstream of *spy49_0661* in both organisms (Fig. 2B). In addition, the MtsR-regulated *nrdF.2IE.2* operon is located adjacent to the homologous gene pair in GBS (Fig. 2A).

### HupY protein binds heme *in vitro*.

**FIG 3:**
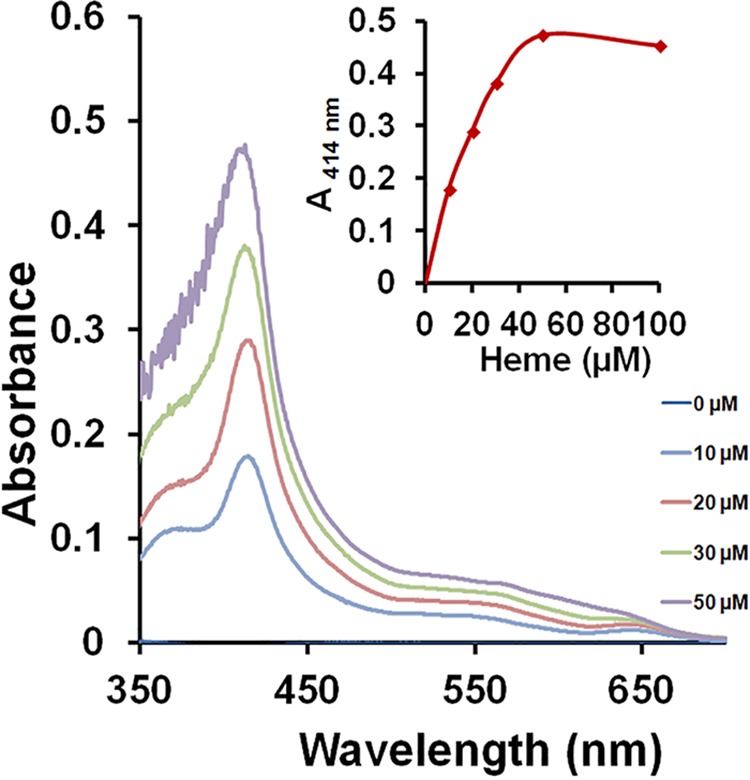
Differential absorption spectroscopy of heme-HupY complex. An increase of heme bound to HupY (17 μM) as increasing concentrations of heme were added to the protein is shown by the sharp peak at 414 nm. The inset displays the changes in absorbance at 414 nm plotted against heme concentration. The data are representative of at least two independent spectroscopic analyses, 0 μM line at axis.

*spy49_0661* had previously been designated *lrrG* in GBS (26) because of its leucine-rich repeat region. GAS and GBS LrrG homologs have been demonstrated to be important adhesins that play a role in infection (26, 27). HupZ can degrade heme and thus may free iron from heme inside the GAS cell (20), but as it is a cytoplasmic protein, it does not have the ability to import heme on its own. To begin testing if Spy49_0661 contributes to heme metabolism in GAS, we cloned and purified the protein. HupY did not exhibit significant absorption other than at 280 nm (Fig. 3, 0 μM heme), indicating that it was purified from E. coli in the apo form. *In vitro* heme binding was tested by titration of the apo protein with increasing amounts of free heme. A growing absorbance band with a maximum at 414 nm appeared in the resultant solution with incremental addition of heme (Fig. 3). The absorption maximum at the Soret region (390 to 430 nm) demonstrated by HupY following incubation with heme is characteristic of protein-bound heme and is distinguishable from that of free heme in solution (20). Plotting the differential absorbance at the Soret peak as a function of heme concentration (Fig. 3) indicated that heme binding was dose dependent, saturable, and with a stoichiometry of 3:1 (protein to heme), indicating that Spy49_0661 can bind heme *in vitro*. We designate this gene *hupY* (heme utilization protein Y) based on its proximity to *hupZ* and its role in heme utilization.

### Δ*hupY* cells have lower intracellular concentrations of iron and impaired ability to use human serum and hemoglobin as sole iron sources compared to those of WT cells.

**FIG 4:**
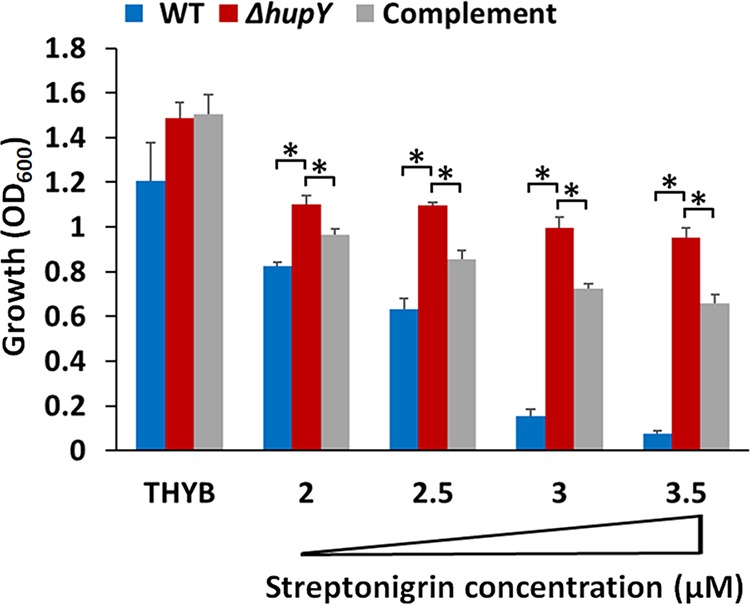
HupY affects intracellular iron concentrations. Shown is growth (20 h) of NZ131 wild-type (blue), Δ*hupY* mutant (red), and complemented (Δ*hupY*/pLC007 [gray]) strains in THYB or in THYB containing streptonigrin. The data are from two independent experiments done in technical triplicates, with SD shown. The asterisk indicates significance (*P* < 0.05, Student’s *t* test, equal variance).

To test the role of HupY in iron metabolism *in vivo*, a deletion of *hupY* was created in GAS strain NZ131. Strains were tested for their ability to resist killing by streptonigrin, an antibacterial whose potency depends on the levels of intracellular iron (17, 33). The *hupY* mutant was able to grow to an optical density at 600 nm (OD_600_) of 1.0 in the presence of high levels (3.5 μM) of streptonigrin. At these concentrations, little to no growth was observed in WT NZ131. The addition of *hupY* on a plasmid under a constitutive promoter was able to partially restore the killing phenotype seen in WT cells (Fig. 4). This indicates that the Δ*hupY* mutant strain has lower intracellular iron concentrations than the WT strain when grown in Todd Hewitt broth supplemented with yeast extract (THYB).

**FIG 5:**
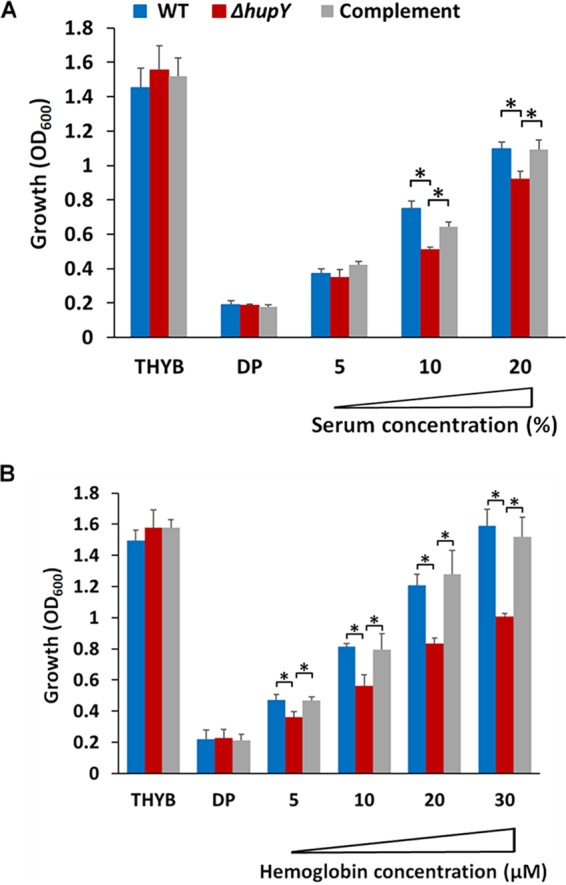
Δ*hupY* mutants are impaired in growth with serum or hemoglobin as the sole iron source. Shown is growth (20 h) of NZ131 wild-type (blue), Δ*hupY* mutant (red), and complemented (Δ*hupY*/pLC007 [gray]) strains in THYB, THYB with dipyridyl (DP), or THYB-DP supplemented with human serum (5 to 20% final volume) (A) or human hemoglobin (B). The data are from two independent experiments done in technical triplicates, with SD shown. The asterisk indicates significance (*P* < 0.05, Student’s *t* test, equal variance).

To test if HupY contributes to the use of heme iron by GAS, THYB was treated with dipyridyl, a liposoluble iron chelator. Human serum or hemoglobin was then added to the medium and the ability of GAS strains to grow in these media was tested. Addition of human serum restored the growth of WT NZ131 in the dipyridyl-containing THYB. This ability was significantly decreased in the Δ*hupY* mutant strain and could be complemented (Fig. 5A). This indicates that the Δ*hupY* strain is impaired in its ability to obtain iron from human serum. Similarly, human hemoglobin was able to support the growth of WT GAS, while the Δ*hupY* mutant had a severe growth defect when growing in hemoglobin as the sole iron source (Fig. 5B).

### HupY plays an important role in vaginal colonization by both GAS and GBS.

**FIG 6:**
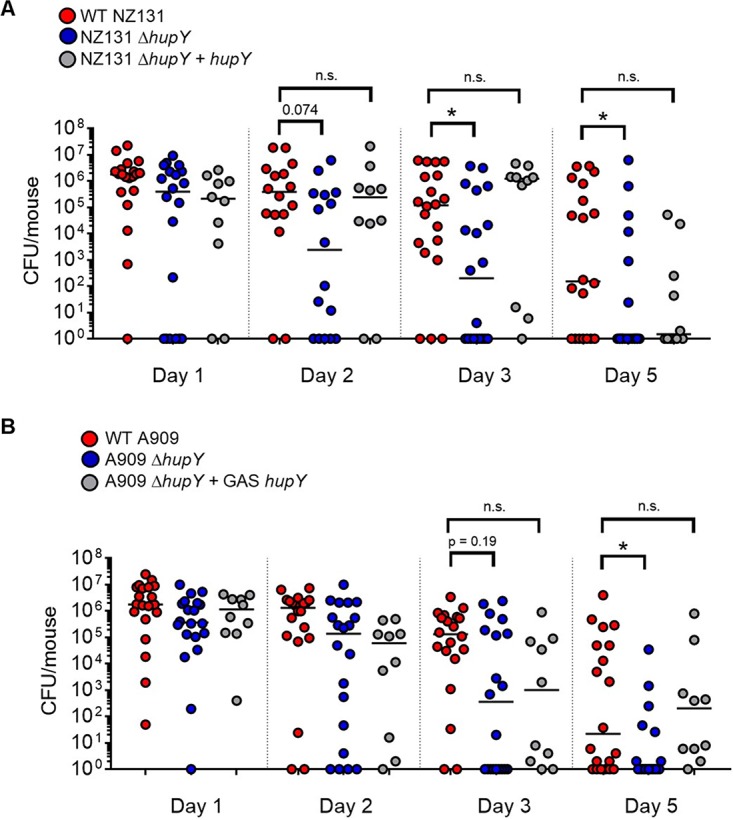
Δ*hupY* mutants in both GAS and GBS are impaired in the ability to colonize the murine vaginal tract. WT and isogenic Δ*hupY* mutants in GAS (A) or GBS (B) were used to colonize the murine vaginal tract. In both cases, the GAS *hupY* gene was used to complement the mutant strains. Both NZ131 Δ*hupY* and A909 Δ*hupY* mutants were attenuated for vaginal colonization compared to WT GAS and GBS, and both mutants were able to be at least partially complemented by the addition of GAS *hupY* on a plasmid. Statistical significance was assessed using a one-way analysis of variance (ANOVA) and nonparametric Kruskal-Wallis test; the asterisk indicates significance (*P* < 0.05). n.s., not significant.

HupY homologs were previously suggested to serve as adhesins with important roles in binding to epithelial cells (26). We hypothesized that HupY would be important for mucosal colonization of the murine vaginal tract. To test this, mice were inoculated with WT NZ131, an isogenic Δ*hupY* mutant, and a mutant strain containing pLC007, a plasmid containing a copy of NZ131 *hupY* under the control of a constitutive promoter. Initial inoculation and day 1 colonization levels were not statistically different between these strains. By day 2 of colonization, the mutant strains were impaired in colonization, and by days 3 and 5, the mutant strains colonized the mice at significantly lower levels than the WT or complemented strains (Fig. 6A).

Because the *hupY* homolog in GBS is closely related and also upregulated in the vaginal tract (16), we hypothesized that this gene would also play a role in colonization of the vaginal tract by GBS. Like GAS, a GBS Δ*hupY* mutant is impaired in colonization of the vaginal tract. The mutant strain was able to be complemented, at least partially, using the GAS *hupY* gene (pLC007), indicating that the heme binding and/or attachment phenotype is likely conserved between GAS and GBS (Fig. 6B).

## DISCUSSION

Bacteria must alter transcriptional programs to survive and adapt to changing environments. In this study, we analyzed the transcriptome of GAS colonizing the murine vaginal tract. These data provide a new and important look at the genetic regulation of various metabolic and virulence pathways in GAS during mucosal carriage. With over one-quarter of the genome differentially expressed during mucosal carriage compared to liquid culture, clearly transcriptional remodeling during colonization is extensive.

Comparing the GAS vaginal transcriptome to the recently published GBS vaginal transcriptome offers much insight into the similarities and differences in the ways these two related organisms adapt to a mucosal environment. While many genes and pathways involved in metabolic processes were similarly regulated in GAS and GBS, there were also many pathways, such as purine biosynthesis, that were upregulated in one species and downregulated in the other (Table S1) (16). These two species obviously share many similarities in terms of adapting to the vaginal environment, but some distinct transcriptional differences are also observed. Comparing transcriptomic profiles from organisms collected from the same model system provides important insight into the differing colonization rates and infections caused by these organisms at the vaginal mucosa.

One striking observation from these data is that many genes in the MtsR regulon are highly activated during GAS colonization of the murine vaginal tract (Table 1). MtsR, an important regulator of metal homeostasis, mediates repression of metal acquisition genes in GAS, including genes involved in heme utilization and iron and manganese transport. Although GBS also contains an MtsR homolog, much less is known about MtsR regulation in GBS. Examining the previously published vaginal transcriptome, homologous genes with potential MtsR binding sites are also derepressed during GBS colonization (Fig. 2) (16).

MtsR was first described as a DtxR family regulator repressing expression of the streptococcal iron acquisition (*sia*) operon in the presence of high levels iron and manganese (17). It was later shown that MtsR controls the expression of 64 genes in GAS, including many that impact iron and heme metabolism. MtsR mutants have been shown to be attenuated for virulence in zebrafish infection models (17), and MtsR has also been shown to affect transcription of important GAS virulence factors, including *mga*, *emm49*, and *ska*, via direct binding to promoter regions (18).

**FIG 7:**
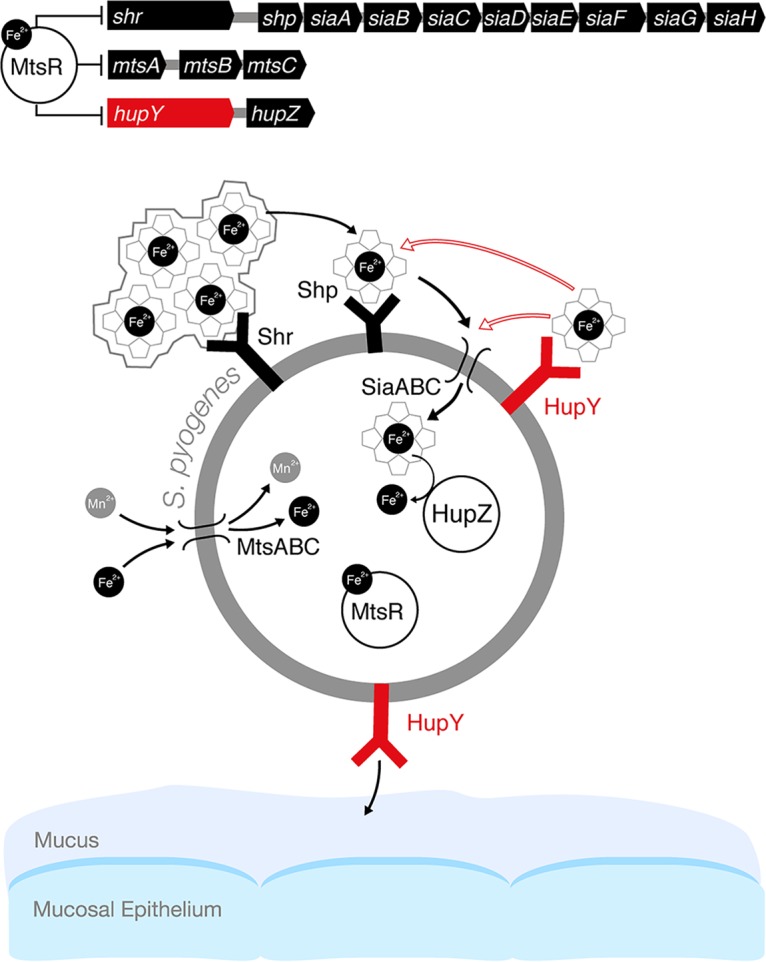
Model of the role of HupY in GAS colonization and heme utilization on mucosal surfaces. In the presence of high levels of iron, iron-bound MtsR represses a large number of genes, including the *sia* operon, which includes the genes for the *siaABC* heme importer as well as *shr*, and *shp* heme binding proteins. In addition, MtsR represses the manganese and iron *mtsABC* transporter, *hupZ*, and *hupY*. MtsR also downregulates the *mtsABC* gene cluster in a manganese-dependent manner (32; not shown in the model). Under iron-depleted conditions such as on mucosal surfaces, MtsR repression is relieved, leading to upregulation of these operons. Shr captures heme (from host hemoproteins or the environment) and delivers it to Shp and subsequently to SiaABC for import into the cell. Once inside the cell, iron can be liberated from heme by the HupZ enzyme. HupY, coregulated with HupZ, is a surface protein that has the ability to bind heme. We propose that HupY binds heme to allow transport to HupZ, either through the Shp/SiaABC heme import pathway or through another mechanism. Open arrows indicate possible heme transfer pathways. HupY also plays a role in mucosal colonization, possibly as an adhesin important for binding to host cells.

Iron is an essential nutrient and heme is a major source of iron for organisms growing in the human host. To use heme as an iron source, bacteria must be able to bind, transport, and utilize the iron present in heme. In GAS, the surface receptor Shr is important for iron uptake, as it binds to heme-containing proteins like hemoglobin and myoglobin and sequesters the heme and transfers it to another surface receptor, Shp, and then to the SiaABC ABC transporter system for internalization (Fig. 7) (17, 23, 34–36). Both Shr and Shp contain NEAT (near iron transporter) heme binding domains found in heme binding proteins of other Gram-positive bacteria, such as the iron-regulated surface determinant (Isd) proteins of Staphylococcus aureus (22). In Shr, NEAT1 and NEAT2 are separated by a leucine-rich repeat domain (23). *shr*, *shp*, and the *siaA* to -*H* genes are all present in a single operon controlled by MtsR and all were found to be derepressed during colonization of the vaginal tract by GAS.

In iron-replete medium, *shr* is expressed more highly during log phase growth in liquid culture and expression is decreased during stationary phase (Z. Eichenbaum, unpublished results). Starving the cells for iron, however, increases further *shr* transcription during log phase due to MtsR depression (18). In our data, *shr* expression is higher on the mucosal surface than during log-phase growth in liquid culture, consistent with iron limitation conditions in the mucosal surfaces. Thus, it is likely that the observed upregulation of these genes is associated with iron availability in the mucosa rather than the difference in growth phase of the two conditions being compared by RNASeq.

*spy49_0661*, here renamed *hupY*, and the gene immediately downstream of it, *hupZ*, are also negatively regulated by MtsR in GAS, and a putative MtsR binding site is found upstream of *hupYZ* in both GAS and GBS (Fig. 2B) (18). *hupYZ* are both significantly upregulated during vaginal colonization by both GAS and GBS (Fig. 2) (16). HupZ was recently described as a novel GAS enzyme that binds heme and is involved in heme biotransformation *in vitro*. Interestingly, HupZ, which belongs to a group of atypical heme-degrading enzymes, does bind and degrade heme, although it is missing key residues found in this protein family (20, 37). As a small cytoplasmic enzyme, HupZ does not appear to be able to transport heme into the cell and would thus rely on other heme binding and transport proteins. Although complementation indicates that the observed phenotypes can be attributed to HupY, because *hupYZ* appear to be under the control of a single promoter, we tested whether the allelic exchange of *hupY* with *aphA3* (Kan^r^) affected expression of *hupZ*. Quantitative reverse transcriptase PCR (qRT-PCR) data showed no significant change in the expression of *hupZ* between the WT GAS or GBS and the Δ*hupY* strains (Fig. S2).

10.1128/mBio.00848-19.2 FIG S2 Expression of *hupZ* is not affected by deletion of *hupY* in GAS or GBS. Quantitative RT-PCR was done on replicate RNA samples collected from WT GAS and GBS strains as well as their isogenic mutant Δ*hupY* strains. Comparison of *hupZ* transcription between the WT and mutant strains showed no significant difference in the expression of *hupZ*, demonstrating that deletion of *hupY* was nonpolar. Download FIG S2, TIF file, 0.03 MB. Copyright © 2019 Cook et al. 2019 Cook et al. This content is distributed under the terms of the Creative Commons Attribution 4.0 International license.

HupY (Spy49_0661 in NZ131 GAS and SAK_0502 in A909 GBS), previously referred to in the literature as LrrG, was initially identified as a leucine-rich repeat protein and putative surface adhesin. The protein sequences of the Spy49_0661 and SAK_0502 homologs in GAS strain NZ131 and GBS strain A909 are 72% identical, both contain an LPXTG motif, and surface localization has been observed in both GAS (27) and GBS (26, 38) homologs. Both have 15 22-amino-acid leucine-rich repeat regions matching the consensus sequence for bacterial leucine-rich repeat proteins (LRRs) (26, 39). The proteins are related to the internalins InlA and InlB from Listeria monocytogenes, which are important for bacterial adhesion to host surfaces via the E-cadherin receptor and subsequent invasion into host cells (40–42). Because of their similarity to internalins and because LRRs are generally involved in protein-protein interactions, previous studies on the LrrG homologs focused mainly on their adherence properties.

SAK_0502 has been shown to bind to fixed HEp-2 lung epithelial cells and ME180 cervical epithelial cells, although with a stronger preference for cervical cells over lung cells (26). Subsequent studies demonstrated binding to the human scavenger receptor gp340 by both GAS and GBS HupY homologs (43). gp340 is expressed by immune cells and epithelial cells, including genital tract cell lines such as VK2E6E7 vaginal epithelial cells and HEC1A endometrial carcinoma cells, and has been shown to be important for HIV transmission in the genital epithelium (44). Based on these data, it is likely that HupY serves an adhesive function in the vaginal tract. HupY homologs have also been proposed as potential vaccine candidates, as they are highly conserved and surface-expressed proteins (26, 27, 43). Immunization against the HupY homologs protected against lethal challenge with GAS, GBS, and Streptococcus equi subsp. *epidemicus* species (26, 27, 45).

Adhesins have been known to have alternative functions, as in the case of the GBS protein Lmb, which is involved in both adherence to human laminin (46) and invasion into brain endothelial cells (47) as well as zinc uptake (48). Similarly, Shr enhances GAS attachment to laminin in addition to its function in heme uptake (49). Based on the genetic relationship with *hupZ* and direct repression by MtsR, we hypothesized that HupY may have dual roles in adherence and heme uptake and degradation as well.

Data presented here show that HupY is able to bind heme *in vitro* (Fig. 3), providing evidence that it may serve as a heme receptor that helps transfer heme inside the cell to HupZ for degradation. In addition, NZ131Δ*hupY* cells are killed less efficiently by streptonigrin, an antibacterial compound that is preferentially toxic to cells with higher levels of intracellular iron, indicating that NZ131 Δ*hupY* cells have less intracellular iron than WT and complemented strains (Fig. 4). This provides evidence that HupY may not only bind heme but also play a role in transporting it into the cell for use as a bacterial iron source. GAS cells were also tested for the ability to grow using hemoglobin or serum as a sole iron source. Δ*hupY* mutants had impaired growth under these conditions, indicating that HupY contributes to the ability of GAS to obtain heme iron from serum and hemoglobin.

Because *hupY* was highly upregulated in the vaginal tract and because of its observed properties as an adhesin, we tested the ability of GAS and GBS Δ*hupY* mutants to colonize the murine vaginal tract. Both GAS and GBS Δ*hupY* mutants were significantly impaired in the ability to colonize the vaginal tract. Complementing the mutation using the GAS *hupY* homolog restored the colonization phenotype in both GAS and GBS, indicating similar functions in the two organisms (Fig. 6). Although it appears that HupY is necessary for vaginal colonization, some questions remain to be answered. We have not yet elucidated whether decreased colonization levels are due to decreased adherence to mucosal surfaces or decreased survival due to an inability to acquire iron from the host. In addition, this mucosal colonization has only been tested in the vaginal tract and remains to be examined at other mucosal sites streptococci are known to colonize, such as the nasopharynx. In GBS the HupY homolog, SAK_0502, was found to bind preferentially to cervical cell lines over lung cell lines (26). It is possible that its role as an adhesin is host mucosal-site specific, while its role in heme binding and utilization is more universal. Further experiments to determine the potential interplay between the adhesion and iron homeostasis functions of HupY are under way.

Unlike heme receptor proteins like Shr and Shp, which contain recognized NEAT domains, HupY does not contain any known heme binding domains. Leucine-rich repeat domains are found in both HupY and Shr and could play a role in heme binding by a novel unknown mechanism. A full analysis of the protein structure and domains involved in binding both the host and heme is an important next step and will allow determination of whether these binding sites are localized to the same regions of the protein.

It also remains to be tested whether HupY plays a role in iron acquisition and utilization in GBS as well as GAS, although all evidence would point to this. The presence of an MtsR binding site upstream of GBS *hupYZ*, the genetic neighborhood of the genes (Fig. 2), and the fact that GAS *hupY* can complement the *in vivo* mutant phenotype in GBS (Fig. 6) strongly indicate that these genes have similar functions in GAS and GBS.

In this study, we have identified a gene, *hupY*, previously thought to encode an adhesin in GAS and GBS, which serves additional functions important for GAS colonization, including heme binding. Both *hupY* and *hupZ* as well as other members of the MtsR regulon are highly derepressed during *in vivo* mucosal colonization in both GAS and GBS. Likely in conjunction with its coregulated neighboring gene, *hupZ*, HupY acts as a heme binding protein involved in regulating intracellular iron levels.

## MATERIALS AND METHODS

### Bacterial strains, media, plasmids, and primers.

Bacterial strains and plasmids used in this work are listed in Table S2, and primer sequences are shown in Table S3. Escherichia coli BH10C was grown in Luria-Bertani medium (LB), and GAS strain NZ131 and its derivatives were grown in Todd-Hewitt medium supplemented with 2% (wt/vol) yeast extract (THY) or chemically defined medium (50). Antibiotics were added at the following concentrations when needed: E. coli, chloramphenicol (Cm) at 10 μg ml^−1^, kanamycin (Kan) at 150 μg ml^−1^, and spectinomycin (Spec) at 100 μg ml^−1^; GAS, Cm at 3 μg ml^−1^, Kan at 150 μg ml^−1^, and spectinomycin (Spec) at 100 μg ml^−1^. Plating of murine lavage fluid was done on CHROMagar StrepB agar plates (DRG; CHROMagar).

10.1128/mBio.00848-19.4 TABLE S2 Bacterial strains and plasmids used. Download Table S2, DOCX file, 0.02 MB. Copyright © 2019 Cook et al. 2019 Cook et al. This content is distributed under the terms of the Creative Commons Attribution 4.0 International license.

10.1128/mBio.00848-19.5 TABLE S3 Cloning and qPCR primers used. Download Table S3, DOCX file, 0.01 MB. Copyright © 2019 Cook et al. 2019 Cook et al. This content is distributed under the terms of the Creative Commons Attribution 4.0 International license.

### Mouse model of vaginal colonization.

Experiments were performed as previously described (14, 51, 52). Female CD1 mice aged 6 to 10 weeks were used for all experiments. Briefly, mice were administered an intraperitoneal (i.p.) injection of 0.5 mg of β-estradiol valerate (Acros Organics) suspended in 100 μl of filter-sterilized sesame oil (Sigma). Intraperitoneal estradiol synchronizes the estrus cycle of the mice. Twenty-four hours later, mice were inoculated with 10^7^ CFU of bacteria in 10 μl of phosphate-buffered saline (PBS) intravaginally using a pipette tip. On days 1, 2, 3, and 5, the vaginal lumen was washed with 50 μl of sterile PBS using a pipette tip to flush the liquid into the vaginal vault 6 to 8 times. Vaginal lavage fluid was then collected and put on ice for no more than 30 min prior to performing serial dilutions in PBS. Dilutions were plated on CHROMagar StrepB plates, which allow the growth of GAS with a purple hue.

### RNA collection.

RNA collection from the murine vaginal tract was done as previously described (16). Fifty microliters of vaginal lavage fluid from 10 colonized mice for 48 h was combined (500 μl total) and put into a tube containing 1 ml of TRIzol LS reagent (Ambion) and placed on ice for no more than 30 min. Samples were centrifuged at 14,000 × *g* for 1 min, and the TRIzol reagent was removed. The Ambion RiboPure bacterial kit (Thermo Fisher; AM1925) was used to purify total RNA from the resulting pellets according to the manufacturer’s protocol. For lysis, the pellets were resuspended in 250 μl of RNAWiz and mixed with glass beads. The samples underwent 10 min of bead beating in a MiniBeadbeater (BioSpec) set to homogenize. DNA was removed from the total purified RNA using DNase I treatment for 30 min at 37°C. For preparation of total RNA from planktonic cultures, bacteria were grown overnight in THY medium overnight. Cultures were diluted 1:50 in freshly prepared CDM medium and grown to an OD_600_ of 0.3 to 0.6. Cultures were centrifuged at the same OD_600_, and RNA was prepared and treated with DNase I as described above for vaginal lavage without the addition of TRIzol LS reagent.

### Depletion of eukaryotic RNA and rRNA from total RNA.

Vaginal total RNA samples were depleted of both eukaryotic RNA and rRNA, and planktonic samples were depleted of only rRNA, as previously described (16). Eukaryotic RNA was depleted using the MICROBEnrich kit (Thermo Fisher; AM1901) and rRNA was depleted using the MicrobExpress kit (Thermo Fisher; AM1905) according to the manufacturer’s instructions. Depletion of eukaryotic and rRNA as well as RNA quality was determined using a TapeStation 2200 (Agilent) and a Qubit RNA high-sensitivity fluorimeter (MBL) by the DNA Services Facility at the University of Illinois at Chicago Center for Genomic Research. If analysis showed remaining high quantities of rRNA, the MicrobExpress kit protocol was repeated to retreat samples.

### Preparation of cDNA libraries for RNASeq.

cDNA RNASeq libraries were prepared as previously described (16). Because of the small amount of input RNA, cDNA libraries were generated with 10 to 400 ng of input RNA using the KAPA stranded RNASeq library (KAPABiosystems; KR0934) according to the manufacturer’s instructions. Briefly, eukaryotic and ribosomally depleted RNA samples were fragmented in fragmentation buffer at 94°C for 6 min. Random primers were used to synthesize the first-strand cDNA before second-strand synthesis, marking, and the addition of a poly(A) tail. Illumina adapters were ligated to the fragments and the subsequent library was cleaned up using DNA purifier magnetic beads (101 Bio; P920-30). The library was amplified for 10 cycles followed by an additional magnetic bead cleanup. Libraries underwent quality control and quantification on the Tapestation 2200 (Agilent) as described above. Twenty microliters of 50 nM libraries were sequenced and analyzed at the University of Chicago Genomic Facility with 8 samples multiplexed per RNASeq lane.

### RNA sequencing analysis.

The quality of reads was assessed using FasQC v0.11.2 (www.bioinformatics.babraham.ac.uk/projects/fastqc/), and reads were mapped to the Streptococcus pyogenes NZ131 genome. Samtools v0.1.19 (30) was used to down-sample all *in vitro* mapped samples. Cufflinks v2.2.1 (31) was used to assemble and quantify transcripts to the NZ131 genome, and assemblies were merged using Cuffmerge and wrapped in nCufflinks v2.2.1. Quantification of transcripts was done using Cuffquant and featureCounts (32). Differentially expressed genes (DEGs) with a false-discovery rate (FDR) corrected *P* value of 0.05 or less and at least a 2-fold change were identified using FPKM (fragments per kilobase per million)-based Cuffdiff and count-based edgeR methods (33).

### Creation of mutant and complemented NZ131 strains. (i) NZ131 Δ*hupY*::*aphA3* strain.

The NZ131 Δ*hupY*::*aphA3* strain was created as described previously (16). Briefly, approximately 1,000 bp upstream (primers LC149 *Pst*/LC150) and downstream (primers LC151/LC152 NotI) of *spy49_0661* (*hupY*) were amplified by PCR. Amplification products were fused in a subsequent PCR using outside primers LC149 PstI and LC152 NotI. The fusion product and temperature-sensitive pJC159 (Cm^r^) vector were digested with PstI and NotI restriction enzymes and ligated together. Using inverse PCR, the plasmid was replicated (primers LC153 MluI/LC154 MluI). The *aphA3* kanamycin resistance gene was amplified from pOSKAR using primers JC292 MluI/JC304 MluI. The resulting linear fragments were digested with MluI, ligated together, and electroporated into competent E. coli BH10C cells. Colonies were screened on LB plates supplemented with Cm and Kan at a permissive temperature (30°C). Plasmid was collected and sequenced to ensure correct assembly and then electroporated into competent NZ131 cells. A temperature-dependent selection process that has been previously described was used to isolate NZ131 Δ*hupY*::*aphA3* cells (53). Cells containing the deletion plasmid were grown at 30°C and then shifted to a higher temperature (37°C) and plated on Cm/Kan THY plates to select for plasmid integration. Following integration, cells were passaged in antibiotic-free THY at 30°C twice daily for 4 days to allow for plasmid excision. Mutants were then selected on THY Kan at 37°C and screened for loss of Cm resistance. Colonies that grew at 30°C in Kan but not Cm were sequenced to confirm the proper genotype.

**(ii) pLC007 complementation plasmid.** The complementation plasmid pLC007 was created by amplifying the *hupY* gene from NZ131 genomic DNA using primers LC184/LC185. Plasmid pJC303, which contains a constitutive *recA* promoter, was digested using NotI and BamHI. The digested plasmid and amplified *hupY* genes were combined in a Gibson assembly reaction using 2× NEBuilder Hifi DNA assembly mix. The assemble plasmid, pLC007, was electroporated into BH10C E. coli cells and plated on LB plates containing spectinomycin. Plasmid was purified, sequenced, and electroporated in competent NZ131 Δ*hupY*::*aphA3* cells and the resulting cells were plated and propagated on THY Kan, Spec plates.

**HupY cloning and purification and heme binding.** The construction of HupY-His expression vectors was accomplished by TOPO directional cloning according to the manufacturer’s instructions (Invitrogen; K101-01). The *hupY* gene was amplified from the GAS NZ131 chromosome using the ZE435/ZE436 primer set (Table S3) and introduced into the pET101/d-TOPO vector (Table S2). The resulting plasmid, pZZ1, codes for a HupY-His tag fusion protein expressed from the T7 RNA polymerase promoter. The recombinant HupY protein was produced and purified from E. coli as previously described (20). Briefly, cells were harvested and resuspended in 20 mM Tris-Cl, 100 mM NaCl, and 0.1% Triton X-100 (pH 8.0), with the addition of 0.5 mg/ml of lysozyme and protease inhibitor (complete mini-EDTA free; Roche), and lysed by sonication. The cell debris was removed by centrifugation and the cleared lysate was applied to a HisTrap HP affinity column (GE Healthcare) and purified using fast protein liquid chromatography (FPLC). Heme titration and differential spectroscopy of HupY-heme complex were conducted as described previously (23). In short, hemin chloride (in dimethyl sulfoxide [DMSO]) was added in increasing concentrations to 17 μM apo-HupY (in PBS), and the solution was allowed to incubate at room temperature for 5 min prior to UV-visible (UV-Vis) scan. PBS with equal amounts of heme served as the blank. The differential UV-visible absorption spectrum was acquired at room temperature with a UV-Vis spectrophotometer (Beckman Coulter; DU730).

### GAS growth in the presence of streptonigrin or using human serum or hemoglobin as an iron source. (i) Streptonigrin killing assays.

Streptonigrin stock solutions (Sigma; 2 mg/ml) were prepared in chloroform and methanol at 1:1 (vol/vol) and kept at −20°C up to 6 months. In each experiment, overnight GAS cultures grown in THYB were used to inoculate fresh broth (OD_600_ of 0.01) containing streptonigrin at various concentrations. The culture optical density was determined after 20 h incubation at 37° C.

**(ii) Serum and hemoglobin use assays.** New 1 M dipyridyl (2,2′ dipyridyl; Acros Organics) prepared in absolute ethanol and 1 mM human hemoglobin (Sigma) prepared in saline (and filter sterilized) were used in all experiments. Fresh THYB containing 3 mM dipyridyl and increasing amounts of pooled normal human serum (Innovative Research) or human hemoglobin were inoculated with overnight GAS cultures grown in THYB (OD_600_ of 0.01). The culture optical density was determined after 20 h of incubation at 37°C. In all experiments, bacteria were grown statically in 6 ml of broth in 15-ml screw-cap Falcon tubes. The WT and Δ*hupY* strains were grown in THYB without antibiotics, as the strains were observed to be stable without selection in antibiotics (unpublished data and Table S2). The complemented strain (Δ*hupY*/pLC007) was cultivated with spectinomycin (100 μg/ml) during the first overnight growth. The presence of the antibiotic markers (kanamycin for the Δ*hupY* mutant and spectinomycin for the complementation plasmid) was confirmed by viable counts using the appropriate antibiotics at the end of each experiment.

### qRT-PCR.

Quantitative reverse transcriptase PCR was done to validate RNASeq data and test the polarity of the Δ*hupY* GAS and GBS mutants on the downstream *hupZ* gene. qPCR primers are listed in Table S3. RNA was collected and treated with DNase I as described above. cDNA was then prepared using the iScript cDNA synthesis kit (Bio-Rad) according to the manufacturer’s instructions. cDNA was amplified using random hexamers and diluted 1:5 prior to qPCR. The *proS* (primer pair LC082/LC083) gene and the *gyrA* (primer pair LC060/061) gene were used as housekeeping reference genes for GAS and GAB, respectively. Amplification was done using iTaq Universal SYBR green Supermix or SSoAdvanced Universal SYBR green Supermix (Bio-Rad) in a CFX96 real-time detection system (Bio-Rad). Samples were run in triplicate technical replicates and at least duplicate biological replicates.

### Ethics statement.

All mouse experimentation was approved by the University of Illinois at Chicago Animal Care and Use Committee (ACC) and IACUC under protocol 16-068. All animal work was carried out using accepted veterinary standards in accordance with the Animal Care Policies of the University of Illinois at Chicago Office of Animal Care and Institutional Biosafety Committee and IACUC. This institution has Animal Welfare Assurance Number A3460.01 on file with the Office of Laboratory Animal Welfare, NIH.

### Data availability.

Sequence data are available as supplemental information (Table S1) and are deposited in the NCBI GEO database under accession no. GSE131982.
