# Supplementary figures and images for: Transcriptomic Analysis of Streptococcus pyogenes Colonizing the Vaginal Mucosa Identifies hupY, an MtsR-Regulated Adhesin Involved in Heme Utilization
Source: mBio. 2019 Jun 25;10(3):e00848-19. doi: 10.1128/mBio.00848-19 (PMC6593403; doi:10.1128/mBio.00848-19)

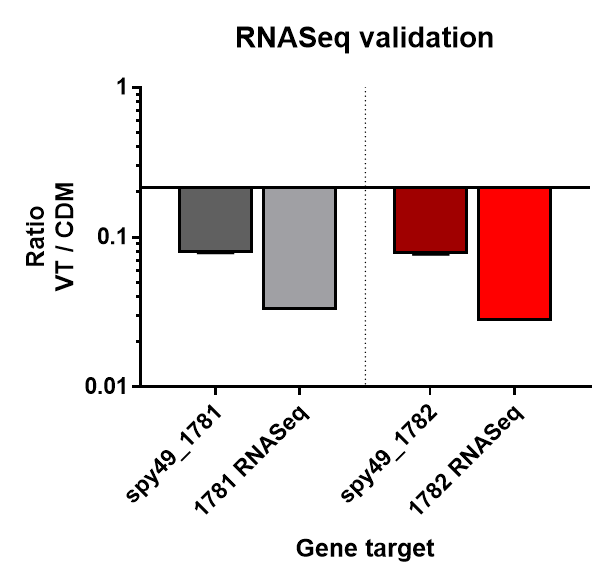

Supplement: FIG S1 [file mBio.00848-19-sf001.tif]

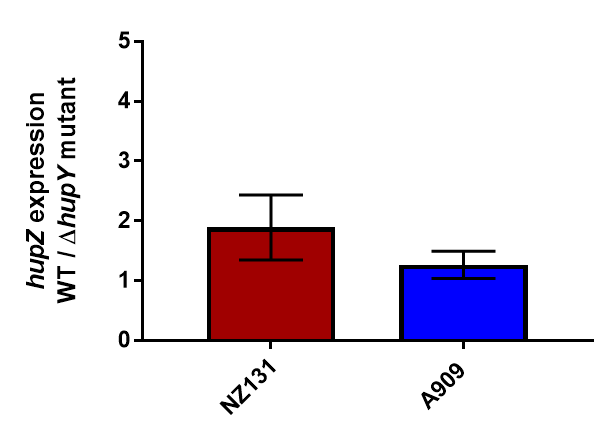

Supplement: FIG S2 [file mBio.00848-19-sf002.tif]
