# Supplementary material for: Transcriptomic Analysis of Streptococcus pyogenes Colonizing the Vaginal Mucosa Identifies hupY, an MtsR-Regulated Adhesin Involved in Heme Utilization
Source: mBio. 2019 Jun 25;10(3):e00848-19. doi: 10.1128/mBio.00848-19 (PMC6593403; doi:10.1128/mBio.00848-19)
Supplement: TABLE S2 [file mBio.00848-19-st002.docx]

**Supplemental data**

**Table S2. Bacterial strains and plasmids used**

| **Strain Name** | **Description** | **Citation** |
| --- | --- | --- |
| NZ131 | Wild-type *Streptococcus pyogenes* strain isolated from a patient with post-streptococcal glomerulonephritis | (1) |
| NZ131*ΔhupY ** | NZ131 *ΔhupY*::*aphA3* Kan^R^ | This study |
| BH10C | *E. coli* cloning strain | (2) |
| E.coli BL21 Star | Host for pZZ1 expression | Invitrogen |
|  | | |
| **Plasmid** | **Description** | **Citation** |
| pFED760 | pGh9-ISS1 deleted for insertion element by inverse PCR; temperature sensitive replication origin, Erm^R^ | (3, 4) |
| pJC159 | pFED760 with *ermB* replaced with chloramphenicol resistance marker *cat*, Cm^R^ | (5) |
| pOsKaR | Source of *aphA3* Kan^R^ cassette | (6) |
| pJC303 | pLZ12-Spec-based vector with *recA* constitutive promoter upstream of multiple cloning site, Spec^R^ | (7) |
| pLC007 ** | *hupY* complementation plasmid, *hupY* inserted downstream of the *recA* promoter in pJC303 | This study |
| pET101/D-TOPO | Directional TOPO^®^TA cloning vector | Invitrogen |
| pZZ1 | pET101 based vector that expresses HupY-His tag from P_T7_ | This study |
| * Mutant is stable and does not require antibiotics for retention  ** Plasmid is retained during overnight growth in liquid culture without the addition of antibiotics | | |

**Supplemental References**

1. Simon D, Ferretti JJ. 1991. Electrotransformation of *Streptococcus pyogenes* with plasmid and linear DNA. FEMS Microbiol Lett 66:219-24.

2. Howell-Adams B, Seifert HS. 2000. Molecular models accounting for the gene conversion reactions mediating gonococcal pilin antigenic variation. Mol Microbiol 37:1146-58.

3. Maguin E, Duwat P, Hege T, Ehrlich D, Gruss A. 1992. New thermosensitive plasmid for gram-positive bacteria. J Bacteriol 174:5633-8.

4. Mashburn-Warren L, Morrison DA, Federle MJ. 2010. A novel double-tryptophan peptide pheromone is conserved in mutans and pyogenic Streptococci and Controls Competence in Streptococcus mutans via an Rgg regulator. Mol Microbiol 78:589-606.

5. Chang JC, LaSarre B, Jimenez JC, Aggarwal C, Federle MJ. 2011. Two Group A Streptococcal Peptide Pheromones Act through Opposing Rgg Regulators to Control Biofilm Development. PLoS Pathog 7.

6. Le Breton Y, McIver KS. 2013. Genetic manipulation of Streptococcus pyogenes (the Group A Streptococcus, GAS). Curr Protoc Microbiol 30.

7. Chang JC, Federle MJ. 2016. PptAB Exports Rgg Quorum-Sensing Peptides in Streptococcus. PLoS One 11:e0168461.
