# Supplementary material for: Transcriptomic Analysis of Streptococcus pyogenes Colonizing the Vaginal Mucosa Identifies hupY, an MtsR-Regulated Adhesin Involved in Heme Utilization
Source: mBio. 2019 Jun 25;10(3):e00848-19. doi: 10.1128/mBio.00848-19 (PMC6593403; doi:10.1128/mBio.00848-19)
Supplement: TABLE S3 [file mBio.00848-19-st003.docx]

**Table S3. Cloning and qPCR primers**

| **Cloning primers** | | |
| --- | --- | --- |
| **Primer** | **Description** | **Sequence** |
| LC149 | *hupY* Upstream S PstI | GACTGCAGAGCTTGTTGACTTAAAGTCAACA |
| LC150 | *hupY* Upstream AS 2step | GTTATCATTTTTTCTCCTTAAATCAATTGTGGTAAAACCTTTCTAAAAGCCAAC |
| LC151 | *hupY* Downstream S 2step | GTTGGCTTTTAGAAAGGTTTTACCACAATTGATTTAAGGAGAAAAAATGATAAC |
| LC152 | *hupY* Downstream AS NotI | AGGCGGCCGCCTTGGATGGTTAGCCATTATCATA |
| LC153 | Plasmid AS MluI inv | ACGCGTATTGATTTAAGGAGAAAAAATGATAAC |
| LC154 | Plasmid S MluI inv | ACGCGTTGTGGTAAAACCTTTCTAAAAGCCAAC |
| LC184 | GAS lrrG compl S Gibson | tgaaaaagattttggtaaggggcggccgcATGAAGAAACATCTTAAAACAG |
| LC185 | GAS lrrG compl AS Gibson | ataacctgaaggaagatctggatccTTAATATTTTTTCTTTTTTATAGCAGTTATTAG |
| ZE435 | *hupY* forward primer, TA cloning | CACCATGCACAATCAGGAAGTTTTT |
| ZE436 | *hupY* revers primer, TA cloning | TATTGCAGAGTGTCGTCCTCTATTCGTTTTT |
|  |  |  |
| **qPCR primers** | | |
| LC060 | *gyrA* S qPCR primer | CAATGGATGGGGATGGTG |
| LC061 | *gyrA* AS qPCR primer | CGCTGGTAAAACAAGAGGTTC |
| LC082 | *proS* S qPCR primer | TGAGTTTATTATGAAAGACGGCTATAG |
| LC083 | *proS* AS qPCR primer | AATAGCTTCGTAAGCTTGACGATAATC |
| LC092 | *spy49_1781* S qPCR primer | TCAAAGAATCCACCCTCTATCC |
| LC093 | *spy49_1781* AS qPCR primer | CCCGAAGAAGTGACAGCATA |
| LC094 | *spy49_1782* S qPCR | AAGGAAGCTGCCAGTGAAAT |
| LC095 | *spy49_1782* AS qPCR | CAGCAATACGGGTAAAGCAA |
| LC192 | *hupY* S qPCR primer | TACTGCAGGTAAAGCGTTGTT |
| LC193 | *hupY* AS qPCR primer | GCCTTCTTGGTCACTAAAGCC |
| LC278 | *hupZ* S qPCR primer | ACAAACCTGTAACCTAAAAG |
| LC279 | *hupZ* AS qPCR primer | AAAGGTCAACCCAATATTGG |
